# Supplementary material for: Time‑resolved multi-omic analysis of paclitaxel exposure in human iPSC‑derived sensory neurons unveils mechanisms of chemotherapy‑induced peripheral neuropathy
Source: Cell Death Dis. 2026 Feb 10;17(1):211. doi: 10.1038/s41419-026-08445-2 (PMC12921266; doi:10.1038/s41419-026-08445-2)
Supplement: Supplementary file 15 — Dataset 1. [file 41419_2026_8445_MOESM15_ESM.zip › CDD_Open_Data/05_Protein/01_Volcano_Plots/VolcanoAndranik/proteomics_volcano.pdf]

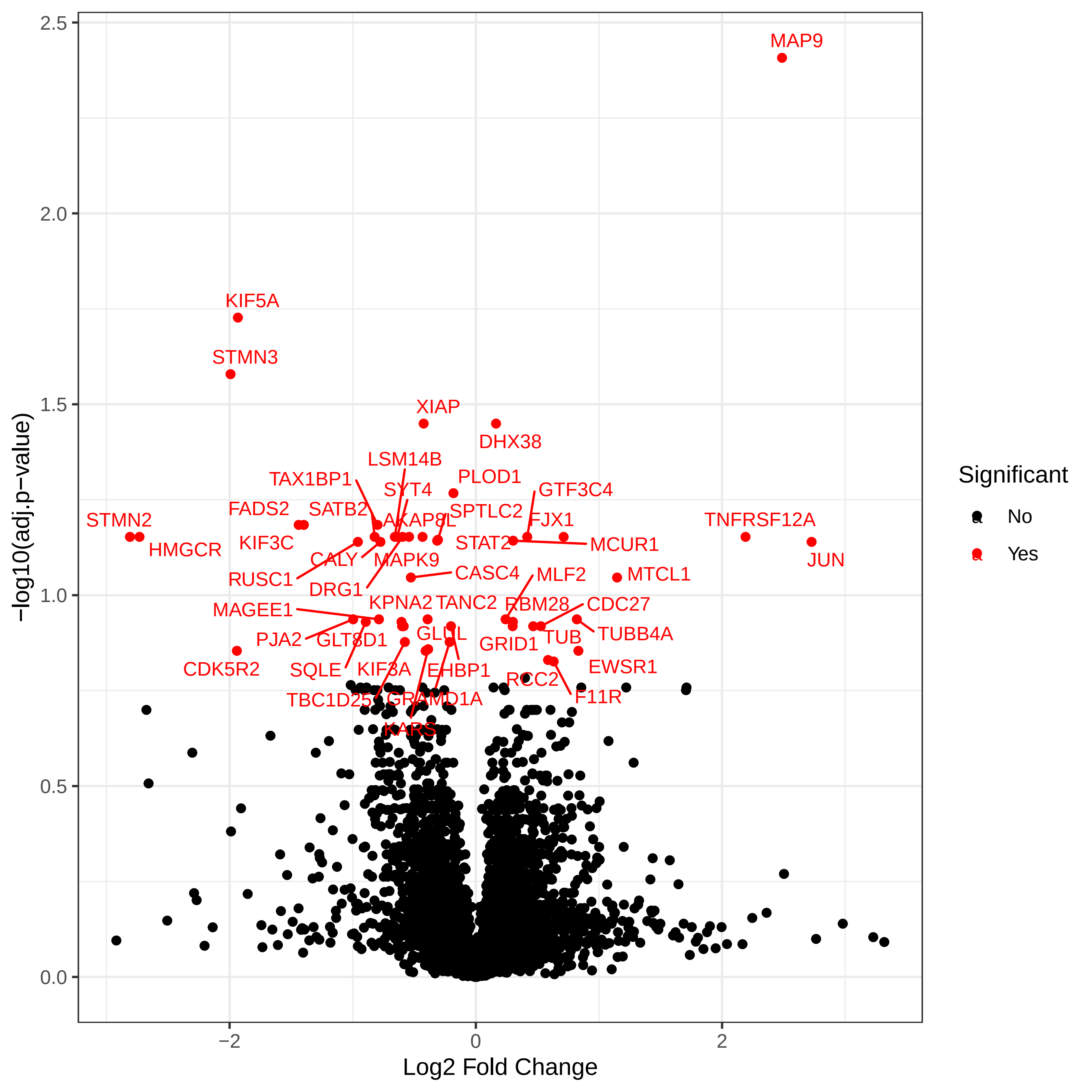

MAP9
KIF5A
STMN3
XIAP
DHX38
LSM14B
TAX1BP1
PLOD1
SYT4
GTF3C4
FADS2
SATB2
SPTLC2
STMN2
AKAP8L
FJX1
TNFRSF12A
HMGCR
KIF3C
STAT2
MCUR1
JUN
CALY
MAPK9
RUSC1
CASC4
MLF2
MTCL1
DRG1
KPNA2
TANC2
RBM28
CDC27
MAGEE1
PJA2
GLT8D1
GLUL
TUB
TUBB4A
GRID1
CDK5R2
SQLE
KIF3A
EHBP1
EWSR1
RCC2
TBC1D25
GRAMD1A
F11R
KARS
Significant
No
Yes
−log10(adj.p−value)
Log2 Fold Change
0.0
0.5
1.0
1.5
2.0
2.5
−2
0
2
